# Bipolar Disorder due to Cushing’s Disease, with manic characteristics. Regarding a clinical case.

**DOI:** 10.1192/j.eurpsy.2024.530

**Published:** 2024-08-27

**Authors:** M. D. C. Blasco Fresco, S. Ciria Villar, L. T. Durán Sandoval, A. Perez Poza, E. De La Fuente Ruiz

**Affiliations:** ^1^Psychiatry, Miguel Servet University Hospital, Zaragoza, Spain

## Abstract

**Introduction:**

The increase in cortisol can be exogenous or endogenous. As etiologies of endogenous increase we find: Cushing’s disease, 68% of cases, generally due to an ACTH-producing pituitary tumor; Adrenal Cushing syndrome (17%); Ectopic Cushing syndrome (15%) due to lung tumor most frequently. It is relevant since among its symptoms one of the most notable are the psychiatric alterations it produces, among them mood disorders, depression being the most common, as well as psychotic symptoms, delirium and anxiety disorder.

**Objectives:**

To carry out a correct differential diagnosis of the pathologies that could present with symptoms of a manic episode.

**Methods:**

Clinical case description of a 52-year-old woman, who presented with manic symptoms in 2020, requiring hospitalization. Upon discharge from the acute care unit, she consulted with the endocrinologist due to weight gain, revealing an increase in abdominal diameter, hyperpigmentation, a moon-like face, and a hump. Free cortisol was measured in 24-hour urine, with a high result, followed by brain MRI, and pituitary microadenoma was confirmed.

**Results:**

The patient underwent surgical resection of the microadenoma, which was partially effective, so she maintained high cortisol levels, even despite oral retreatment. In 2023 she had a new manic episode, with a cortisol value of approximately 300 nmol/day.

**Conclusions:**

The importance lies in the correct diagnosis to provide appropriate treatment and avoid the chronicity of the disease and the patient psychiatrization. In this case and as in many other diseases, which present with psychiatric symptoms, it is important to differentiate whether it is a primary psychiatric disorder or are component symptoms of another disease that, upon receiving treatment, would resolve the psychiatric symptoms.

**Disclosure of Interest:**

None Declared

